# Intrathecal Infusion of Autologous Adipose-Derived Regenerative Cells in Autoimmune Refractory Epilepsy: Evaluation of Safety and Efficacy

**DOI:** 10.1155/2020/7104243

**Published:** 2020-01-03

**Authors:** Elzbieta Szczepanik, Hanna Mierzewska, Dorota Antczak-Marach, Anna Figiel-Dabrowska, Iwona Terczynska, Jolanta Tryfon, Natalia Krzesniak, Bartlomiej Henryk Noszczyk, Ewa Sawicka, Krystyna Domanska-Janik, Anna Sarnowska

**Affiliations:** ^1^Clinic of Child and Adolescent Neurology, Institute of Mother and Child, Poland; ^2^Mossakowski Medical Research Centre, Polish Academy of Sciences, Poland; ^3^Department of Plastic Surgery Centre of Postgraduate Medical Education, Poland; ^4^Clinic of Child and Adolescent Surgery, Institute of Mother and Child, Poland

## Abstract

*Objective/Purpose*. Evaluation of efficacy and safety of autologous adipose-derived regenerative cells (ADRCs) treatment in autoimmune refractory epilepsy. *Patients*. Six patients with proven or probable autoimmune refractory epilepsy (2 with Rasmussen encephalitis, 2 with antineuronal autoantibodies in serum, and 2 with possible FIRES) were included in the project with approval of the Bioethics Committee.

**Method:**

Intrathecal injection of autologous ADRC acquired through liposuction followed by enzymatic isolation was performed. The procedure was repeated 3 times every 3 months with each patient. Neurological status, brain MRI, cognitive function, and antiepileptic effect were monitored during 12 months.

**Results:**

Immediately after the procedure, all patients were in good condition. In some cases, transient mildly elevated body temperature, pain in regions of liposuction, and slight increasing number of seizures during 24 hours were observed. During the next months, some improvements in school, social functioning, and manual performance were observed in all patients. One patient has been seizure free up to the end of trial. In other patients, frequency of seizures was different: from reduced number to the lack of improvement (3-year follow-up).

**Conclusion:**

Autologous ADRC therapy may emerge as a promising option for some patients with autoimmune refractory epilepsy. Based on our trial and other clinical data, the therapy appears to be safe and feasible. Antiepileptic efficacy proved to be various; however, some abilities improved in all children. No signs of psychomotor regression were observed during the first year following the treatment.

## 1. Introduction

### 1.1. Background

Epilepsy is a chronic neurological disorder diagnosed in 1% of the population, nearly 400,000 patients in Poland. About 70% of these patients respond positively to antiepileptic therapy, and 30% of people with epilepsy remain refractory to any administered pharmacological and biological treatment [1]. Frequent epileptic seizures lead to cognitive deterioration and patient social isolation.

The findings over the recent years suggest that among the patients with drug-resistant epilepsy, the group with autoimmune factors may constitute a significant part, often without full features of limbic encephalitis [2, 3]. The majority of them was found to have an antineural antibody. Except humoral immunity, cell response may play a pathogenic role, for example, in Rasmussen's encephalitis [4]. In addition, clinical features, e.g., multiple types of seizures or faciobrachial dystonic seizures, resistance to antiseizure drugs, personal or family history of autoimmunity, history of recent or past neoplasia, and evidence of CNS inflammation, may suggest the autoimmune background of the disease. For these patients, when standard immunomodulative therapies (intravenous immunoglobulins, steroids, immunosuppressants) are ineffective or severe side effects appear that limit their application, stem cell-based therapy may turn out to be a “hope” therapy.

Over the past few years, it has been possible to observe an increasing number of ongoing clinical trials involving the application of mesenchymal autologous or allogenic stem cells (MSC). Clinical trials with adipose-derived regenerative cells (ADRCs) have only commenced a few years ago, and since then, 80 clinical trials have been started. Up today, the remarkable effectiveness of ADRC therapy has been demonstrated mostly in diseases with autoimmune background such as *systemic lupus erythematosus* (*SLE*), rheumatoid arthritis, human graft-versus-host disease, or Crohn's disease. These protective and anti-inflammatory properties are strictly connected with direct effect of MSC on immune cells. Several groups demonstrated that MSC inhibit different effector functions on immune cell populations including T and B cells, dendritic cells (DC), and natural killer cells. MSC are thought to promote survival of T cells in quiescent state [5], inhibiting their proliferation not through apoptosis but through cell division arrest [6]. Other groups showed that mesenchymal stem cells inhibit B cell proliferation, immunoglobulin production, and B cell differentiation to antibody-secreting cells [7]. The belief in a positive effect of ADRCs in drug-resistant epilepsy comes from abovementioned and described ability of mesenchymal cells to migrate and settle in the injured tissue, but mostly due to their strong immunomodulative, especially anti-inflammatory, properties.

However, the protective properties of ADRCs depend strictly on their stage-related differentiation determining, among other, their paracrine activity. Our data gathered in preclinical and clinical experiments indicate that freshly isolated, undifferentiated, and highly proliferating MSC are the most effective in various adjuvant-based therapeutic approaches [8]. With extended culture, the cells differentiate and their protective effect declines in parallel with changes in their paracrine/adjuvant capabilities.

Mesenchymal stem cells derived from adipose tissue are of particular interest due to the only moderate level of invasiveness of the isolation procedure, as well as comparatively high efficiency per number of cells obtained. Even if the pure population of stem/progenitor cells in fact make up less than 5% of all total ADRCs, it still occurs in a frequency about 2500 times higher than found in standard bone marrow aspirate (0.002%). Thus, in the majority of cases, this “technical” property allows them to be collected directly from adipose tissue via liposuction eliminating the need for cell culture [9, 10].

The isolated heterogeneous fractions of adipose-derived regenerative cells (ADRCs) have been proven to possess functional trophic and immunomodulatory properties [11], with capacity to support multidirectional cell differentiation [12]. The pre- and clinical data suggest that ADRCs improve blood flow, modulate the inflammatory response, and protect compromised tissues from risk of further cell degeneration and elimination. Furthermore, compared with MSC derived from other sources, ADRCs show greater plasticity, longer survival times, and higher vasculogenetic and lineage differentiation potential especially after the cell engineering. ADRC can be induced to differentiate into osteocytes or chondrocytes but also under defined inductive environment into muscle, endothelial, or neuroectodermal cell lineages [13]. Interestingly, in such inductive environment, they augment secretion of numerous angiogenesis-related and trophic cytokines, such as vascular endothelial growth factor (VEGF), placental growth factor (PGF), and transforming growth factor *β* (TGF-*β*), as well as ensuring moderate expression of fibroblast growth factor 2 (FGF-2) and angiopoietin 1 (ANG-1). Due to such properties, it is believed that these cells can be very promising when applied across multiple degenerative conditions possessing ischemic etiological component.

Patients with drug-resistant epilepsy exhibit a decreased number of neurons in epileptic zone, with the rest of neurons being hyperactive and malfunctioning. Such a typical, postinjury regions are well visible in the affected brain tissue and increase after epileptic seizure. The employed ADRC treatment may inhibit local inflammation and induce activation of endogenous neurogenesis and angiogenesis which may compete effectively with this ischemia and inflammation-dependent neurodegeneration. In experimental model of epilepsy (kainate-induced neuron damage), MSC treatment can rescue hippocampal neurons from apoptosis. Moreover, MSC-derived soluble factors protect CNS neurons against glutamate excitotoxicity neurons [14]. It has been demonstrated that ADRCs show strong neuroprotective, immunomodulating, and antiapoptotic properties, potentially reducing the frequency of epilepsy seizures [15, 16]. Thus, taking together, clinical trials with ADRC addressed to drug-resistant epilepsy patients would be promising in controlling the epileptic seizures and coexisting behavioral/psychiatric symptoms.

The goal of our project was to evaluate effects of ADRC therapy, including its safety and efficacy in patients with autoimmune drug-resistant epilepsy. Moreover, we tried to correlate the clinical data with properties of isolated and injected cells. The new method in addition to the traditional pharmacological ones would bring hope to reduce the incidence of seizures, to improve the patients cognitive and social functioning which should lead to overall improvement in the overall quality of their lives.

### 1.2. Study Design

**Scheme 1:**
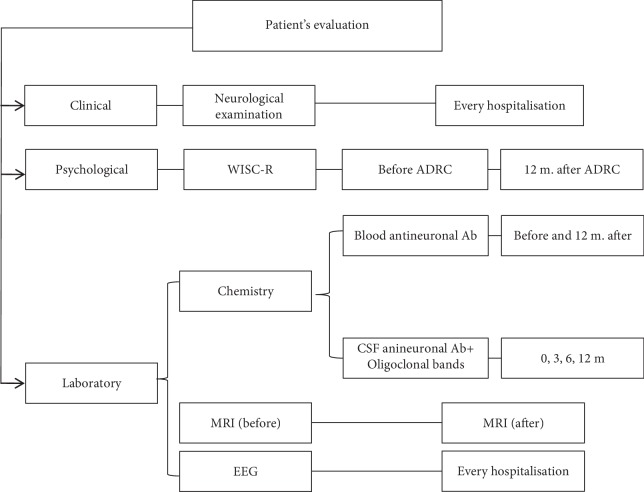
Scheme of patients' evaluation. The diagram presents the applied neurological and electrophysiological methods and psychological and biochemical assessment, along with their assignment to time of assessment.

The study was registered with NCT03676569. It was designed as a nonrandomized, prospective, single-center, open-label study, with no placebo control, to assess safety and efficacy of ADRC transplantation into the children with refractory epilepsy caused by an autoimmune mechanisms. Patients who were diagnosed with autoimmune drug-resistant epilepsy and have met the set criteria were qualified to take part in the further examination. After a formal written consent of their parents, at the Clinic of Child Neurology IMC, the patients underwent neurological examination, routine laboratory tests, EEG, neuropsychological assessment, and then ADRC intrathecal injections 3 times every 3 months. Safety, adverse events, and efficacy were confirmed by neurological status, brain MRI, cognitive function, and antiepileptic effect during 24 months. Both the study protocol (Scheme 1) and the informed consent procedure were approved by the Bioethics Committee of the Institute of Mother and Child in Warsaw. Prior to enrolment to the study, each participant was given detailed “Information for the study participant” and signed an informed consent in 2 copies, one for each party.

### 1.3. Patients

According to inclusion and exclusion criteria (Table 1), a group of potentially eligible patients was identified and included in the project: 6 patients (2 boys, 4 girls) with mean age of 10 years and with proven or probable autoimmune refractory epilepsy: 2 with Rasmussen encephalitis, 2 with neuronal autoantibodies, and 2 with possible febrile infection-related epilepsy syndrome (FIRES). All patients had normal birth parameters, and their development was in normal range up to refractory epilepsy onset. Apart of patients with Rasmussen syndrome, their MRIs did not show any abnormalities. Extended metabolic investigation did not detect any inherited disorders including GLUT1 deficiency.

Patients' basic medical and psychological data are shown in Table 2.

#### 1.3.1. Case Number 1 (P1)

There was a 16.5-year-old boy whose epilepsy started at the age of 7 years. From the beginning of the disease, he had left-sided or general tonic-clonic seizures. Brain MRI showed atrophy of the right hemisphere characteristic of Rasmussen syndrome. He was treated with oxcarbazepine (OXC), carbamazepine (CBZ), valproic acid (VPA), levetiracetam (LEV), clobazam (CLB), and topiramate (TPM) in various combinations but without significant effect. Neurosurgical undercutting of the motor cortex was also performed without clear effect. From the age of 10, left-sided progressive hemiparesis started. In serum, anti-LGI-1 antibodies and oligoclonal bands were detected. Corticoid pulses and immunoglobulin infusions were added to antiepileptic drugs but only with short transient improvement. Due to frequent seizures without drug response, tacrolimus was administrated at the age of 14 with clear but transient effect. His family has a medical history of autoimmune disorders. He demonstrated IQ in the low average score with significant visuospatial deficits relevant to right hemisphere lesion.

#### 1.3.2. Case Number 2 (P2)

A 16-year-old girl with epilepsia partialis continua was diagnosed when she was 6 years old. Her seizures were always left sided. MRI showed mild atrophy in the right hemisphere, the clinical picture suggestive for Rasmussen syndrome. Several antiepileptic drugs were administrated: VPA, OXC, CNZ (clonazepamum), PHT (phenytoin), and STM (sultiamum) but without result. Additionally, steroid pulses and immunoglobulin infusions were applied. From the age of 11, left-sided progressive hemiparesis started. Antineuronal antibodies anti-AMPA and anti-LGI-1 in serum were detected. Tacrolimus was added to therapy for 6 months. As a result, no antineuronal antibodies were detected, although there was no improvement in seizure frequency. Her IQ was in the average score with predominance of verbal abilities. Anxiety and depression symptoms were also periodically observed secondary to her health problem.

#### 1.3.3. Case Number 3 (P3)

There was an 8.5-year-old boy, whose epilepsy with polymorphic seizures began in the age of 1.5 years. He was treated by several drugs such as VPA, CBZ, LEV, and TPM in various drug combinations followed by Synacthen, getting a temporary annual remission at the age of 3 y, but developed transient cushingoid obesity. At the age of 4, epilepsy recurred and antineuronal anti-LGI-1 antibody was detected in serum. Corticoid pulses and intravenous infusions of immunoglobulin were added to antiepileptic drugs with only transient improvement. The addition of immunosuppressant Imuran caused marrow aplasia and the treatment was stopped.

His IQ is in the low average score. Significant visuomotor deficits, attention, and learning problems were observed.

#### 1.3.4. Case Number 4 (P4)

There was a 12-year-old girl, whose epilepsy with polymorphic seizures started at the age of 3. She was treated by several drugs in various combination of VPA, LEV, LTG, LEV, ETS (petinimid), TPM, CLB, CNZ PHT, and NTZ (nitrazepam), with only transient effect. At the age of 6, antineuronal anti-AMPA and anti-GFAP antibodies were detected in serum. At the beginning, her EEG showed generalized abnormal changes followed by hypsarrhythmia. Her IQ showed regression to moderate mental retardation.

#### 1.3.5. Case Number 5 (P5)

There was a 7-year-old girl with refractory focal epilepsy from the time she was 4.5 years old, which started after febrile infection. Various combinations of antiepileptic drugs were administered, VPA, PB (phenobarbital), TPM, VGB, LEV, and CBM. A FIRES was diagnosed. Mild hypo-IgA and IgM immunoglobulinemia was detected before the start of immunomodulatory therapy. Immunoglobulin infusions followed by steroid pulses were applied without clear improvement. At the age of 5.5, tacrolimus was administrated with periodic remission of seizures. Her IQ is in the average score, but she had attention and memory disorders.

#### 1.3.6. Case Number 6 (P6)

There was a 12-year-old girl with family history of autoimmune disorders, whose refractory focal epilepsy started at the age of 9 years following febrile infection. Previously, she was diagnosed with hypothyreosis. She was treated with VPA, CNZ, DZM (diazepam), CBZ, OXC, CLB, and PHT, in various combinations without results. Diagnosis of FIRES has been made. Steroid pulses and immunoglobulin infusion were administrated with only short transient remission. Tacrolimus was also used for 1 year with short improvement. Her IQ is in the low average score with deficits in verbal skills. Symptoms of transient aphasia were observed during increasing frequency of seizures.

### 1.4. Patient Evaluation Scheme

**Figure 1:**
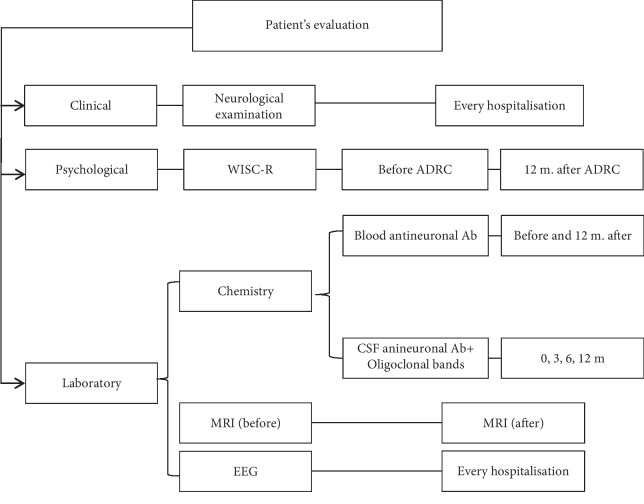
EEG of patient no 3. (a, b) EEG before ARDC treatment (Nov. 2015): spike and spike-slow wave complex: continuous on the left temporal and centro-parieto-occipital region and frequent on the right centro-parietal region. (c) EEG after the last ARDC treatment (Apr. 2016): sharp waves and spikes: frequent on the left temporal and centro-parieto-occipital region and a few on the right centro-parietal region.

The patients' evaluation was divided into three sections: neurological status, cognitive function, and laboratory tests (Figure 1) and lasted 12 months. Each clinical observation was done during a 5-day hospitalization at IMC, including prevention and treatment of potential side effects. Although experimental clinical trial was settled for 12 months to assess the effectiveness of the therapy, the observation was extended to 3 years to assess the safety of the therapy.

Brain MRI was performed on a 1.5 T unit apparatus. SE/T1-, FSE, T2-weighted, and FLAIR images were obtained in axial, coronal, and sagittal planes.

Neuropsychological assessment (cognitive evaluation) was performed by Wechsler Intelligence Scale for Children-Revised (WISC-R) for children aged 6-16 years yield an intelligence quotient, which includes subsets assessing verbal skills, attention, visuospatial skills, and arithmetic abilities.

The protein level, oligoclonal bands, IgG index, and GluR3, VGKC complex/LGI1, GM1, NT-3, GAD, and NMDAR antibodies in cerebrospinal fluid (CSF) were analyzed in 4 time points: before cell therapy, 3, 6, and 12 months after the first ADRC injection.

Ongoing patient observation was carried out in the outpatient conditions in order to assess frequency of epileptic seizures and the overall health condition—based on information provided by the patients and their relatives. A checkup after 3, 6, and 12 months was done.

## 2. Materials and Methods

### 2.1. Surgical Procedures

#### 2.1.1. Liposuction

The subcutaneous adipose fat tissue was harvested from the abdomen or inner thigh parts after infiltration with Klein solution (based on lactated Ringers solution with 6.5 ml 1% lidocaine and 0.5 mg epinephrine per 500 ml lactated Ringers) under local anaesthesia. In order to preserve regenerative properties of cells, fat collection was performed in low pressure, using syringe liposuction. We obtained approximately 250-100 ml of lipoaspirate during each procedure using 1-2 mm harvesting cannulas.

#### 2.1.2. ADRC Isolation

Under operating room conditions, immediately after collection, the lipoaspirate was processed in the CellCelution 800 System (Cytori Therapeutics Inc., San Diego, CA). It was washed to remove free blood and lipids and digested with Celase 800 (Cytori Therapeutics Inc.) enzyme to release the stromal vascular fraction. After a series of centrifugation steps and passing through a system of sieves with various pores, the stromal fraction was concentrated, and 5 ml of ADRC suspended in Ringer solution was prepared for transplantation: 4 ml was intended for patient's treatment, while 1 ml of remnant stromal vascular fraction was used for *in vitro* analysis.

#### 2.1.3. Intrathecal Stem Cell Injection

Intrathecal stem cell injections were performed through lumbar puncture. Lumbar needle was placed at the level of the L3 or L4 vertebrae, so that the introducing needle entered below the level at which the spinal cord ended. First, 5 ml of cerebrospinal fluid was collected for protein level, oligoclonal bands, IgG index, and GluR3, VGKC complex/LGI1, GM1, NT-3, GAD, and NMDAR antibody analysis. Next, through the same cannula, cell suspension (4 ml) was injected over 2 minutes followed by saline injection (1 ml over 30 sec). From each derived ADRC sample, 1 ml was collected for further biochemical and biological analysis.

### 2.2. Cell Analysis

Preclinical characteristic of ADRC according to ISCT guideline and additionally rate of proliferation (PDT), genetic stability under 5% oxygen, and maintenance of stemness properties together with commitment to cell differentiation toward mesodermal direction have been done and published [28].

### 2.3. Flow Cytometry Analysis

To detach cells from the culture dishes, Accutase Cell Detachment Solution (Becton Dickinson) was used. Centrifuged cells (1 × 106) required for the analysis were suspended in a cold stain buffer (Becton Dickinson). Using Human MSC Analysis Kit (Becton Dickinson), according to manufacturer's protocol, to each tube was added the appropriate dilution of fluorochrome-conjugated antibodies directed against APC CD73, FITC CD90, and PerCP-Cy*™*5.5 CD105 (positive markers) and PE CD34, PE CD11b, PE CD19, PE CD45, and PE HLA-DR (negative markers) and incubated for 30 minutes at room temperature, protected from light. After incubation, cells were washed twice with stain buffer, suspended in 500 *μ*l of this buffer and immediately analyzed using FACSCalibur II cytometer (Becton Dickinson). 10,000 events were counted using FACSDiva software computer program.

### 2.4. CFU-F Assay

To assess the capacity and efficiency for self-renewal, freshly isolated cells were seeded on 6-well plates at a density 100 cells/well and cultured in standard culture conditions for the next 10 days. Day 11 cultures were fixed with 4% PFA RT 15′ and stained with 0.5% toluidine blue for 20 minutes in 35% ethanol. Stained colonies made up of more than 50 cells were scored as CFU and were counted. Calculation of the CFU-F efficiency was performed according to the known formula: CFU − F efficiency = (number of colonies/cells originally seeded) × 100.

### 2.5. Secretory Properties of ADRC in the Presence of Patients CSF

ADRC and CSF (before cell injection) were collected from each patient. When ADRC culture reached semiconfluency, medium was changed to autologous CSF (300 *μ*l/well) for 48 hours. After that time, CSF was collected and stored in -80°C. CSF samples after coculture with ADRC were analysed using Luminex kit as described below (2.4.4) to assess the secretory properties of cells in response to an inflammatory factors presented in patients' CSF.

### 2.6. Determination of Chemokine and Cytokine Profile

**Figure 2:**
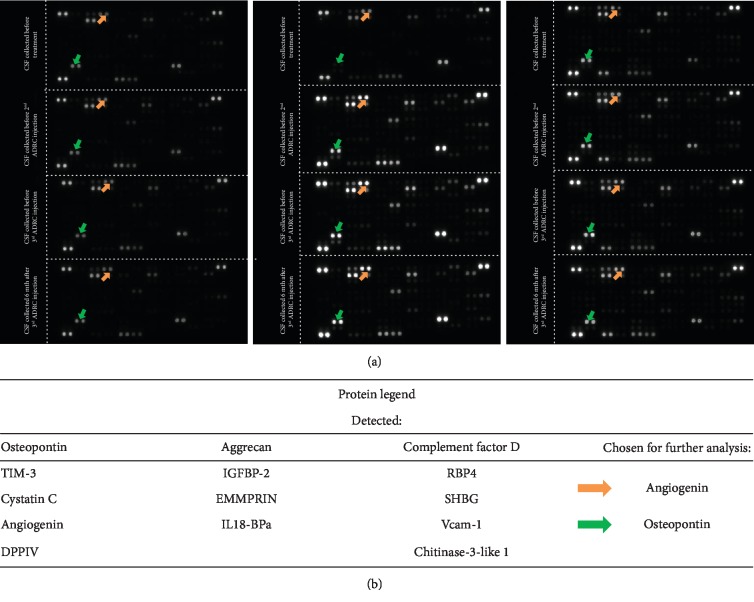
Qualitative assessment of cytokines in CSF. (a) Membrane-based antibody arrays for the parallel determination of the relative levels of human cytokines and chemokines from CSF of patients with epilepsy (from left side: P3, P4, and P6). (b) Table—detected and chosen analytes for luminex multiplex cytokine analysis method.

The qualitative analysis of 102 chemokines or cytokines was performed for CSF samples using the Proteome Profiler Human XL Cytokine Array Kit (R&D Systems) following the producer's protocol (Table 3). The membranes were incubated overnight with 300 *μ*l of each sample and then washed and incubated with the detection cocktail, with streptavidin-conjugated horseradish peroxidase (R&D Systems) and finally with the chemiluminescence reagent. The spots were visualized by Fusion FX6 (Vilber Lourmat) with EvolutionCapt FX6. The registered signals were proportional to the amount of the bound analyte (Figure 2).

### 2.7. Cytokine and Chemokine Assays with Luminex Kit

A Bio-Plex 200™ System (Bio-Plex® 200 Systems, USA) and 6-plex Human Magnetic Luminex Assays (R&D Systems, cat. no. LXSAHM-06) were used to measure the cytokine or protein concentration in CSF samples. The analytes included Osteopontin, Angiogenin, IL-10, TNF-alpha, and CXCL12/SDF-1. The concentration values were obtained using Luminex 200 IS V2.1 Software. Standard curves were generated from the reference cytokine gradient concentrations. All samples were prepared in this same way; after CSF collection, samples were immediately transported in 2-8°C to the laboratory, divided into a smaller portions and stored in -80°C. All procedures with CSF analysis were conducted on the ice. Each sample was frozen/thawed only once.

### 2.8. Statistical Analysis

Recorded data were statistically analysed using a GraphPad Prism 5^th^ version software (La Jolla, CA, USA). The mean ± SEM was calculated for all sample; the significance was determined using Student's *t*-test or one-way ANOVA followed by adequate post hoc test. The values were considered as significant with a value of *p* < 0.05 (^∗^*p* < 0.05, ^∗∗^*p* < 0.01, ^∗∗∗^*p* < 0.001, and ^∗∗∗∗^*p* < 0.0001).

## 3. Results

### 3.1. Clinical Outcome

#### 3.1.1. Seizure Frequency, Side Effects, and Neuroimaging

One patient (P3) with 100 seizures/day before the treatment has been seizure-free up today (responder). He had no side effects of therapy. Mild or transient improvement in seizures was observed in two patients (P1 and P6—50% reduction of seizures persists up today), and these patients were called mild responders. In P4, the transient improvement was observed only after the first ADRC infusion. In the last one, P5, any improvement was observed. P4 and P5 were described as nonresponders. The EEG record of only P3 showed transient improvement after 3-4 months (Figure 1).

All patients except one had mild side effects such as bruises and pain in place of liposuction or febrile 1-2-day reactions. Two children (P1 and P4) had an increase in the number of seizures during second and third days after the first transplantations (Table 4).

All children except one, who had transient mild hypogammaglobulinemia, probably caused by immunosuppressive therapy before transplantation, had normal results of immunoglobulins and testes assessing cellular immunity (LTT: lymphocyte transformation test and cytometry).

MRI examinations did not show any changes compared to the previous state.

#### 3.1.2. Cognitive Functions

**Figure 3:**
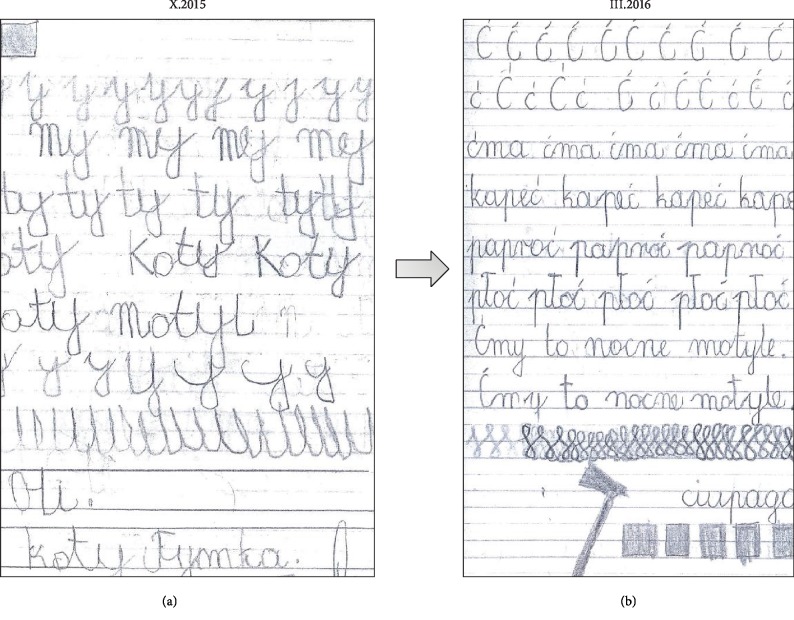
Photos of patient's notebook. (a) Before the first ADRC infusion. (b) 5 months after ADRC infusion—manual ability improvement is visible.

In psychological examination, no changes in the IQ or neuropsychological deficits were found. However, improvement of the general functioning—general interest, psychic drive, attention durability, cognitive performance, and general activity—was noted. Significant improvement in writing and drawing skills was observed in P3 (Figure 3). Additionally, any signs of psychomotor regression were observed one year after the end of treatment.

#### 3.1.3. Intrathecal IgG Synthesis

**Figure 4:**
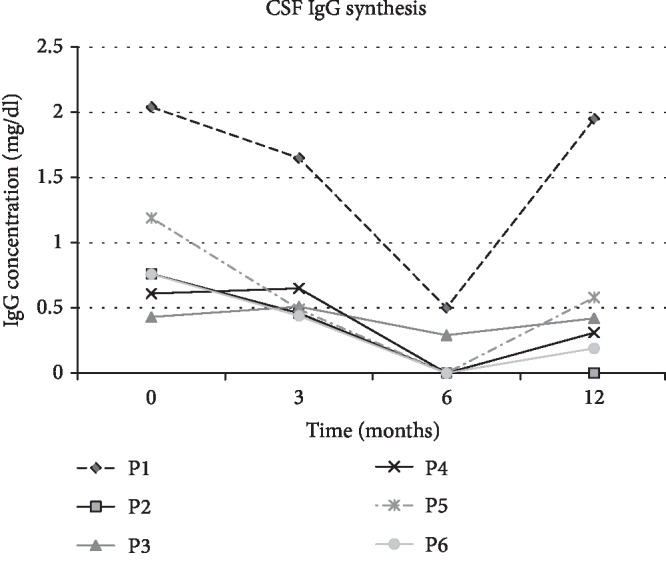
Intrathecal IgG synthesis. Gradual decrease in intrathecal synthesis of IgG after a 3- and 6-month ADRC treatment, followed by an increase in the next 6 months since the last administration; *N* < 0.01 mg/dl, P1-P6—resp., patient 1–6.

Intrathecal IgG synthesis was elevated in all patients before ADRC treatment. Additionally, one patient had positive oligoclonal bands in CSF. After ADRC infusion, decrease in IgG synthesis was observed in all patients with minimal value of 6 months after the first infusion. Reduction degree of IgG level did not correlate with seizure frequency. Unfortunately, 12 months after treatment (6 months after the last injection), tendency to increase IgG synthesis was observed again (Figure 4).

#### 3.1.4. Cytokine and Chemokine Profile in CSF

In order to correlate the course of the disease with the factors secreted by injected cells, 102 cytokines/chemokines have been analysed in the CSF samples of our patients before and after cell treatment (Materials and Methods). For qualitative analysis, proteome profiler was used. The difference in the concentration (over 10%) was noticed for Aggrecan, Angiogenin, Chitinase-3-like 1, Complement factor D, Cystatin C, DPPIV, EMMPRIN, IGFBP-2, IL18-BPa, Osteopontin, RBP4, SHBG, TIM-3, and vCAM-1 (Figure 2(a)).

Based on the above results and possible involvement in epileptogenesis, we have chosen Angiogenin and Osteopontin (Figure 2(b)) for further quantitative analysis. Additionally, IL-10, TNF-alpha, and CXCL12/SDF-1 alpha were added to the analysis.

**Figure 5:**
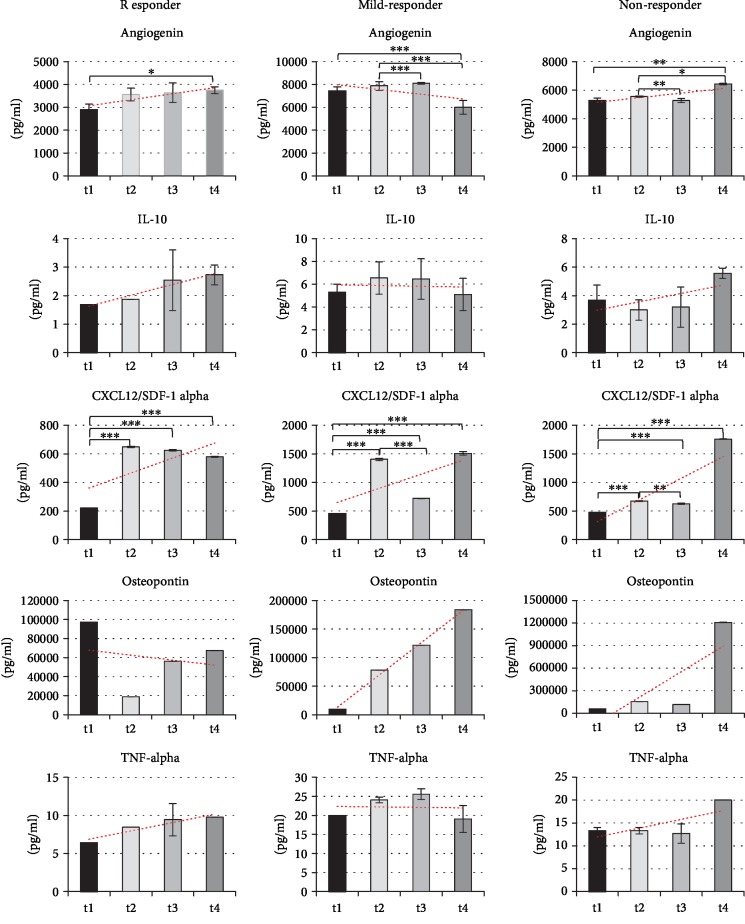
Quantitative analysis of cytokine and chemokine level in CSF during ADRC treatment. Comparison of Angiogenin, IL-10, CXCL10/SDF-1*α*, Osteopontin, and TNF-*α* concentration, determined by the Luminex multiplex cytokine analysis method, in CSF, before and after ADRC application (black graph bars/t1—before ADRC application, light grey bars/t2—3 months after 1^st^ ADRC application, medium grey bars/t3—3 months after 2^nd^ ADRC application, dark grey bars/t4—6 months after 3^rd^ ADRC application, P3—respond patient number 3, P6—mild responder, P4—nonresponder.

In quantitative analysis (Figure 5), Angiogenin, CXCL12/SF1alpha, and Il-10 level in CSF increased in the following months after cell therapy and did not differ responder from nonresponder. The factor that significantly differs between them was Osteopontin. In CSF derived from responder patient, Osteopontin concentration significantly decreased after the first ADRC application (approximately 5 times lower) from very high starting level (10 × 10^4^ pg/ml) and maintained at the low level during the whole time of experimental therapy. In the mild and nonresponders, its concentration increased in the each analysed time point (Figure 5).

#### 3.1.5. ADRC Analysis In Vitro

To find the factors responsible for different therapeutic effects of cell therapy, 1 ml of ADRC suspension, remained after each clinical application, was analysed *in vitro*. The results from the three selected patients (according to clinical data: responder, mild responder, and nonresponder) were presented.

#### 3.1.6. MSC Phenotype

**Figure 6:**
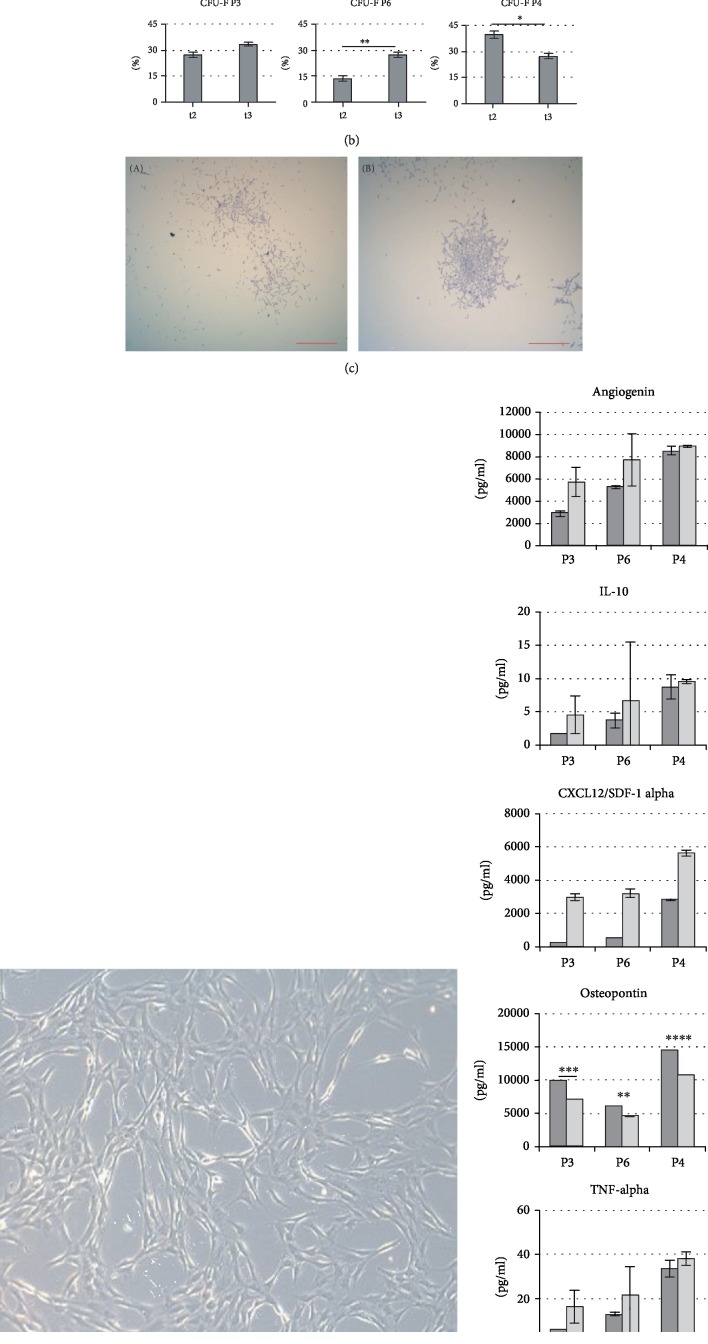
ADRC *in vitro* analysis. (a) Flow cytometry analysis—surface markers analysis of ADRC. (b) Quantification of CFU frequency—P3: responder, P4: mild responder, P6: nonresponder, t2: ADRC isolated after 2^nd^ liposuction, t3: ADRC isolated after 3^rd^ liposuction. (c) CFU morphology from P3: (A) paraclone; (B) meroclone-like structures, scale bar—500 *μ*M. (d) The morphology of ADRC derived from P3, scale bar—100 *μ*M. (e) Secretory properties of ADRC exposed to patients CSF: Luminex multiplex cytokine analysis method. Dark grey bars: control, 48 h after incubation with CSF aspirated from responder (P3), mild responder (P6), and nonresponder (P4) before therapy.

ADRC isolated from all patients expressed typical for MSC features, required by ISCT (International Society for Cellular Therapy), e.g., fibroblast-like morphology (Figure 5(d)), mesodermal-line differentiation, clonogenic potential, and determined pattern of surface antigens (data not shown, Lech et al., 2016). Also typical for MSC surface markers (CD90-99.8%, CD73-99.7%, CD105-99.6%, and CD34, CD11b, CD19, CD45, and HLA-DR in 0.1% of cells) were confirmed (Figure 6(a)).

#### 3.1.7. Clonogenic Potential

The CFU-F (colony-forming unit fibroblast frequency) in responder (P3) and mild responder increased with each subsequent liposuction, and in P3 was 27% and 33%, respectively, in mild responder (P6) 14% and 28%, respectively (Figure 6(b)). In nonresponder (P4), CFU-F decreased from 44% to 18%, respectively.

Most of colony-forming unit presented morphology typical for paraclone-like colonies (consisted mainly of loosely packed, enlarged cells with a mean density, but unusually with large diameter) and meroclones with irregular outline, smaller size in diameter, consisting of small more tightly packed cells and larger loosely packed cells around the edge. The third type of colony structure, similar to holoclones, which consist of small and the most tightly packed cells, was not noticed (Figure 6(c)).

#### 3.1.8. The Influence of CSF on ADRC Secretory Properties *In Vitro*

ADRCs were cultured in CSF collected from responder, nonresponder, and mild responder before cell therapy. Secretion of Angiogenin, Il-10, CXCL/SDF1*α*, and TNF-*α* increased in each case, but apparently in cells exposed to responder CSF. There was an increase of Angiogenin under the influence of responder CSF of 50%, while in nonresponder and mild responder increased 28% and 12%, respectively. IL-10 in responder increased 80%, and in mild and nonresponder 47% and 12%, respectively. The level of CXCL/SDF1*α* increased 97% in responder CSF, and in mild and nonresponder 84% and 47%, respectively. An increase in TNF-*α* concentration in responder was 87%, while in mild responder 61% and nonresponder only 8%. Only Osteopontin concentration significantly decreased after ADRC exposure to patients' CSF, in responder CSF to 60%, in mild responder to 83%, and in nonresponder to 73% (Figure 6(e)).

## 4. Discussion

In some patients suffering from refractory epilepsy, disorders in the humoral and cellular immune responses that could be responsible for the pathogenesis of seizures are described. In these group of patients, pharmacological treatment (antiepileptic drugs) and other therapeutic approaches are often not effective. Persistence of seizures is accompanied by a significant progressive impairment of cognitive functions and quality of life, and disturbed social life. The above problems were observed in all our patients. Worse functioning was manifested as apathy, lack of daily activity, reticence, and gradual intellectual regression.

According to literature, the medical history and diagnostic tests, in refractory epilepsy with autoimmune background, in about 10-20% of patients changes in the levels of immunoglobulins (e.g., IgA in serum) have been described. Among our patients, one of them had a medical history of IgM and IgA-class hypoimmunoglobulinaemia. In patients with autoimmune epilepsy, changes in the peripheral lymphocyte population are also described. The number of Th4 helper lymphocytes is usually lower, while the number of Th8 suppressor lymphocytes increases, NK (natural killer) cells number decreases, and cytokine production by mononuclear cells is stopped. Increased production of proinflammatory cytokines, i.e., interleukin- (IL-) 1*α*, IL-1*β*, and IL-6, has been found in serum and cerebrospinal fluid [17].

The criteria for the diagnosis of autoimmune epilepsy apart from the presence of an antineural antibodies in serum and CSF also include the presence of other antibodies against specific neuron components in the serum, e.g., against monosialoganglioside (GM1), sphingomyelin ganglioside (LM1), glutamate receptor fragment (GluR3), or against glutamic acid decarboxylase [18]. Abovementioned antineural antibodies were detected in our patients serum or CSF (Table 2).

Patients with autoimmune epilepsy usually respond well to immunomodulatory treatment, i.e., infusion of immunoglobulins, steroid pulses, or immunosuppressive treatment. Adverse effects occurring during the administration of high doses of immunoglobulins (0.4 g/kg/body weight/day) are rare, e.g., suppression of the immune response. After immunomodulative therapy, nearly 30% of the patients are completely seizure free, while approximately 40% experience a significant, but temporary, clinical improvement, as well as an improvement in electroencephalographic examination. High efficiency is also achieved after treatment with steroids or immunosuppressants, i.e., azathioprine or tacrolimus. Unfortunately, this treatment carries a risk of serious side effects, i.e., marrow aplasia as we saw in our patient number 3 (P3).

Examples of syndromes that manifest by drug-resistant seizures, cognitive deterioration, and autoimmune background are Rasmussen-type brain inflammation (P1 and P2) and FIRES P5 and P6.

Due to the lack of antiepileptic drugs effect in our group of patients and occurrence of serious side effects after immunosuppressive therapy that enabled the continuation of such aggressive pharmacological treatment, we decided to start experimental MSC application.

Our results demonstrate that in a 3-year follow-up, ADRC treatment is feasible, safe, and quite well tolerated by pediatric patients. Severe adverse reactions to the administration of the cells were not observed. Only transient fever or pain within the liposuction area were observed. Similar mild adverse after MSC intrathecal injections were described by other groups [19].

Unfortunately, only one of six patients (P3) achieved full remission (no seizures for more than 3 years), three of six had mild and transient seizure frequency reduction and two of six had no effect. This is much less optimistic result compared to the other groups. Hlebokazov et al. described 80% of patients as responders, including 30% patients with remission [20]. The possible explanation of our study result may be long-lasting epilepsy with numerous seizures in our patients resulting in neurodegeneration of cerebral tissue.

The patient free of seizures after the first ADRC injection (P3) was the only one that had in his medical history marrow aplasia after immunosuppressants and before MSC injection. This factor could be taken under the consideration as positive factor for future MSC therapy. Unexpectedly for us in his case the decrease in intrathecal IgG synthesis was insignificant. Moreover, in the other patients, the decrease of IgG intrathecal synthesis is not always correlated with seizure reduction. We consider that the greatest decrease in immunoglobulin levels 9 months after AD-MSC administration may reflect a transient decrease in inflammation in the brain. Unfortunately, after 12 months, immunoglobulin levels began to increase again, which was probably associated with the gradual depletion of the cells given and thereby the cessation of their actions.

The anti-inflammatory and immunomodulatory effects of AD-MSC may lead to diminishing the neuroinflammation process in the brain and might reduce seizure activity. But, autoimmune inflammatory processes in the brain of our patients had been long lasting and very complex and probably to eliminate them completely, longer and more sophisticated therapy should be needed. Moreover, the underlying immune-related pathology and chronic neuroinflammation process lead to neurodegeneration which is hardly reversible and is responsible for refractory epilepsy.

When analyzing *in vitro* ADRC isolated from his adipose tissue, we found that they formed proliferation centers with the best morphology: small, packed cells with round or spindle shape (Figure 6), although the number of CFU-F was comparable to that of the nonresponder.

The only nonquestioned positive effect described in all patients after cell therapy was an improvement in daily functioning (Figure 1), e.g., greater activity and willingness to communicate. At the same time, in neuropsychological tests, no significant cognitive improvement was noted. Therefore, we associate this effect with mood improved (antidepressant effect) and increased propulsion. Over the last three years, we have not found further developmental regression in any of our patients. Shwartz's group described similar effect in animal model of depression and connected that with increasing both glutamate uptake and neurotropic factor secretion in the hippocampus [21]. The possible mechanism involved in positive ADRC effect including remission or transient seizure reduction is similar to that in other autoimmune diseases, i.e., lupus erythema or rheumatoid arthritis—immunomodulation and stabilization of the pathological systemic status due to adjuvant MSC properties. The effects of implanted cells are exerted via soluble humoral factors and regulatory immune cell conversion. Such anti-inflammatory effect may be transient due to limited cell survival what we [22, 23] and others [20] have previously reported. MSC after transplantation are thought to retain for 2 to 3 months, which makes necessary to repeat applications but increases the safety of therapy. The limited cell survival affected the intrathecal synthesis of antineuronal antibodies, which decreased during therapy but started to increase again after the end of in 5 of 6 patients (Figure 4).

As long as ADRCs are present in the cerebrospinal fluid, they produce wide range of anti-inflammatory cytokines and chemokines as well as growth factors. Immunomodulatory MSC effects were observed in other clinical trials. Kwon et al. have shown that IL-10, TGF-*β*, and IL-6 level were increased in the CSF after intrathecal MSCs injection. The authors explained that intrathecal MSC injection caused the peripheral immune cell infiltration into the patient's CNS and conversion of these cells toward regulatory T lymphocytes and Th2 lymphocytes, which in turn release anti-inflammatory cytokines such as IL-4, IL-10, and TGF-*β* [24]. Other group described after dual (i.v. and intrathecal) MSC therapy the downregulation of activated lymphocytes and antigen-presenting cells, resulting in a 30–60% reduction in CD86+, CD83+, HLA-DR+ myeloid dendritic cells, and activated CD40+ cells, and a 72% increase in CD4+ and CD25+ regulatory T cells [25]. Additional described antiepileptic effects of injected MSC could be related to neuroprotection, through reduced loss of GABAergic interneurons, low level of myeloperoxidase, and anti-inflammatory genes encoding cytokines in the hippocampus; upregulation was also reported [26].

In our patients, among the 102 cytokines analyzed in CSF, only the level of 5 changed significantly: Angiogenin 1, IL-10, stromal cell-derived factor 1 (CXCL12/SDF-1), Osteopontin, and TNF-*α*.

Osteopontin was the only one that differs between responder and nonresponders. Its concentration decreased in responder, most significantly after the first ADRC administration, what correlated with the lack of seizures from that time. The role of OPN in neurodegenerative diseases causes widespread concern. OPN is likely to be an effective therapeutic target for neurodegenerative diseases, but simultaneously, other researcher groups show its involvement in the pathogenesis processes. Osteopontin has been reported to be upregulated after injury, ischemia [30], kainate-induced seizures [31], or experimental autoimmune encephalomyelitis [32]. However, in mentioned diseases, its effect was assessed in two ways. Osteopontin was described to have a neuroprotective effect in ischemic injury in the brain [33], but in contrast, it was reported to promote inflammation and exacerbate autoimmune diseases. Osteopontin is upregulated also in response to the epileptic seizures. The other mentioned cyto- and chemokines play a significant role in epileptogenesis.

Angiogenin (ANG) is the multifunctional factor that plays role in angiogenesis but also in autoimmune diseases as anti-inflammatory agent. ANG reduces the mRNA expression of interleukin-1 beta (IL-1*β*), IL-6, IL-8, and TNF-*α* receptors (TNFR) 1 and 2 and factor-alpha (TNF-*α*) and increases the mRNA expression of IL-4 and IL-10 [34].

IL-10, the anti-inflammatory agent, is significantly downregulated in patients with febrile seizures (FS) relatively to normal controls [35, 36]. The low level of IL-10 in CSF may play a role in the etiopathogenesis of FS. Furthermore, IL-10 has been found to provide direct trophic and prosurvival cues to neurons during development. It was suggested that exogenous administration of IL-10 may have direct beneficial effects on neurons [37]. Increased level of the anti-inflammatory IL-10 could be the consequence of ANG induction or may be the result of direct secretion of activated by inflammatory environment of MSC.

TNF-*α* has one of the pivotal roles in epileptogenesis. Its level increases after each seizure. The anti-TNF-*α* therapy for epilepsy was considered although was not applied due to the suspected risks of infection and cancer development [38]. TNF-*α* growth may be associated with transient inflammation caused by MSC injection, but on the other hand, its elevation increases MSC migration to injured tissue [39].

Except the secretory properties, MSC migratory ability toward damaged or chronically irritated regions is important. For this purpose, the stromal cell-derived factor 1 (SDF-1/CXCL12) chemokine receptors CXCR4 and CXCR7 should be activated in the damaged tissue. The reduced SDF-1 expression results in a decreased ASC migration. SDF-1/CXCR4 and SDF-1/CXCR7 pathways seem to play an important role in the brain plasticity, adult neurogenesis in the DG, and regulation of GABA release in synaptic area. Overall, this chemokine system could be one of the key players of the neuroimmune environment [40].

We observed also that ADRC in response to factors present in patients' CSF increased their secretory properties. The most significant cell activation was observed after exposure to CSF drown from responder. The mechanisms underlying ADRC response to CSF could depend from, e.g., to the duration of the disease (inflammatory factors present in CSF), secondary neurodegenerative process, genetic background, or previous history of pharmacological immunomodulatory treatment.

Although the ability of MSCs to transdifferentiate *in vitro* into neural cell-like types was demonstrated by our and other teams [27–29], cell replacement *in vivo* is still uncertain. The limited 3-month survival of MSC transplanted into CSF [22] excludes rather restorative theory by now.

Since we presented only a small single-center study, the data presented here are rather clinical observations than the results of a clinical trial. Low patient number has not allowed us to create statistically significant recommendations; rather, they are a guide for further research.

## 5. Conclusions

Therapy with ADRC in autoimmune refractory epilepsy seems to be safe and feasible. Unfortunately, most likely only a small group of patients with autoimmune etiology will benefit from such therapy, but we cannot exclude that the result of MSC therapy would be better when applied at early point in the disease progression. We still do not know the factors that allow us to predict which patients will be the responders. It is possible that marrow aplasia could predispose to better outcomes. ADRC injection does not change permanently EEG, especially when prolonged seizures led to focal neurodegeneration. However, MSC therapy improves general functioning of these patients. Considering the changes in the cytokine level in CSF, it seems that Osteopontin may be the best biomarker and predictor of the outcome from therapy while changes in IgG intrathecal synthesis do not correspond with the results of treatment. Allogenic transplantation for MSC therapy in autoimmune diseases might be considered in future therapies.

## Figures and Tables

**Table 1:** Inclusion and exclusion criteria for the intrathecal ADRC treatment.

Inclusion criteria	Exclusion criteria
(i) Presence of antineuronal antibody in serum or CSF	(i) Refractory epilepsy with proven genetic or metabolic etiology
(ii) Rasmussen encephalitis (proven cellular immunity pathogenesis)	(ii) Neoplastic process
(iii) Probable autoimmune pathogenesis (autoimmune diseases in family, FIRES—febrile infection-related epilepsy syndrome)	(iii) Status epilepticus
(iv) Patient's or parents' agreement	(iv) Infection

**Table 2:** Basic medical and psychological data.

Patient no.	Age	IQ	Diagnosis	Disease onset (yrs)	Duration of refractory epilepsy (yrs)	MRI	Antineuronal antibody
1	16.5	82	RS	9	7	Marked right hemisphere atrophy	Oligoclonal bands
2	16	110	RS	6	9	Mild right hemisphere atrophy	Anti-AMPA1, anti-LGI1
3	8.5	87	ARE	1.5	7	Normal	Anti-LGI1
4	12	41	ARE	3	9	Normal	Anti-AMPA1
5	12	80	FIRES	9	4	Normal	No
6	7	113	FIRES	4.5	3.5	Normal	No

ARE: autoimmune refractory epilepsy; ADRCs: adipose-derived regenerative cells; LGI1: leucine-rich glioma inactivated 1; FIRES: febrile infection-related epilepsy syndrome; RS: Rasmussen encephalitis; IQ: intelligence quotidian; No: no detected. Antibodies to receptors: AMPA1-alpha 1 glutamate ionotropic receptor.

**Table 3:** Cytokine array assay.

Cytokines, chemokines, and acute phase proteins		
Adiponectin/Acrp30	INF-gamma	Lipocalin-2/NGAL
Aggrecan	IGFBP-2	CCL2/MCP-1
Angiogenin	IGFBP-3	CCL7/MCP-3
Angiopoietin-1	IL-1 alpha/IL-1F1	M-CSF
Angiopoietin-2	IL-1 beta/IL-1F2	MIF
BAFF/BLyS/TNFSF13B	IL-1ra/IL-1F3	CXCL9/MIG
BDNF	IL-2	CCL3/CCL4 MIP-1 alpha/beta
CD14	IL-3	CCL20/MIP-3 alpha
CD30	IL-4	CCL19/MIP-3 beta
CD40 ligand/TNFSF5	IL-5	MMP-9
Chitinase 3-like	IL-6	Myeloperoxidase
Complement component C5/C5a	IL-8	Osteopontin (OPN)
Complement factor D	IL-10	PDGF-AA
C-reactive protein (CRP)	IL-11	PDGF-AB/BB
Cripto-1	IL-12 p70	Pentraxin 3/TSF-14
Cystatin C	IL-13	CXCL4/PF4
Dkk-1	IL-15	RAGE
DPPIV/CD26	IL-16	CCL5/RANTES
EGF	IL-17A	RBP4
CXCL5/ENA-78	IL-18 BPa	Relaxin-2
Endoglin/CD105	IL-19	Resistin
EMMPRIN	IL-22	CXCL12/SDF-1 alpha
Fas ligand	IL-23	Serpin E1/PAI-1
FGF basic	IL-24	SHBG
KGF/FGF-7	IL-27	ST2/IL1 R4
FGF-19	IL-31	CCL17/TARC
Fit-3 ligand	IL-32 alpha/beta/gamma	TFF3
G-CSF	IL-33	TfR
GDF-15	IL-34	TGF-alpha
GM-CSF	CXCL10/IP-10	Thrombospondin-1
CXCL1/GRO alpha	CXCL11/I-TAC	TNF-alpha
Growth hormone (GH)	Kallikrein 3/PSA	uPAR
HGF	Leptin	VEGF
ICAM-1/CD54	LIF	Vitamin D BP

**Table 4:** Epileptic seizure characteristic before and after treatment, EEG evaluation, and drug without changes.

Patient	Before experimental therapy			After experimental therapy		
	Epileptic seizure character	Drugs	EEG	Epileptic seizure character	EEG	Improvement
Patient 1	Left sided generalizing, GTCS, polimorphic	VPA, CBZ, LEV, TPM (tacrolimus)	Simple changes, lateralized on the right side	Left sided, polimorphic	Simple changes, generalized and localized in the left fronto-central region	Transient reduction in number and severity of attacks
Patient 2	Left sided continuous	VPA, CBZ, PHT, OXC, (tacrolimus)	Frequent changes localized in right fronto-central region	Left sided continuous	Very frequent, bilateral changes localized in fronto-centro-pariertal region, more on the right	Mild reduction of gait disturbances
Patient 3	GTC, myoclonic	VPA, LEV, TPM, CBZ, (azathioprine)	Continuous changes localized predominantly in the left temporal and centro-parieto-occipital region	No seizures	Frequent changes localized predominantly in the left temporal and centro-parieto-occipital region	100% improvement in functioning, lack of seizures
Patient 4	Polymorphic (atypical absences, GTCS, myoclonic)	VPA, PHT, CBZ, TPM, (azathioprine)	Frequent generalized and bilateral changes localized in the fronto-central region, more on the right side, hypsarrhythmia-like	Polymorphic (atypical absences, GTCS, myoclonic)	Very frequent generalized and bilateral changes localized in the fronto-central region, more on the right side, background slowing	Transient improvement after the first ADRC infusion
Patient 5	Focal with/without impartment of consciousness (GTCS)	VPA, CBZ, TPM, CLB	Frequent changes lateralized on the right side	Focal with/without impartment of consciousness, GTCS	Frequent changes lateralized on the right side	No
Patient 6	Focal with/without impartment of consciousness, GTCS	VPA, PB, LEV, TPM, VGB	Frequent changes localized in the left parasagittal region and simple changes localized in the fronto-central region on the right side	Focal with/without impartment of consciousness, GTCS	Frequent changes localized in the parasagittal region bilaterally more on the left side	50% improvement in number and severity of attacks

EEG: electroencephalography; VPA: valproic acid; CBZ: carbamazepine; LEV: levetiracetam; TPM: topiramate; PHT: phenytoin; OXC: oxcarbazepine; CBZ: clobazam; PB: phenobarbitalum; VGB: vigabatrin; GTCS: generalized tonic-clonic seizures.

## Data Availability

The data from clinical study used to support the findings of this study are available from Prof. Elzbieta Szczepanik (elzbieta.szczepanik@wp.pl) and from basic study from the corresponding author upon request.
